# miR-19a/b-3p promotes inflammation during cerebral ischemia/reperfusion injury via SIRT1/FoxO3/SPHK1 pathway

**DOI:** 10.1186/s12974-021-02172-5

**Published:** 2021-05-29

**Authors:** Feng Zhou, Yu-Kai Wang, Cheng-Guo Zhang, Bing-Yi Wu

**Affiliations:** 1 Research Center of Clinical Medicine, Nanfang Hospital, Southern Medical University, Guangzhou, 510515 Guangdong Province, People’s Republic of China; 2 Department of Neurology, First People’s Hospital of Foshan, Foshan, 528000 Guangdong Province, People’s Republic of China

**Keywords:** Ischemia, miR-19a/b-3p, SIRT1, FoxO3, SPHK1, Inflammation

## Abstract

**Background:**

Stroke affects 3–4% of adults and kills numerous people each year. Recovering blood flow with minimal reperfusion-induced injury is crucial. However, the mechanisms underlying reperfusion-induced injury, particularly inflammation, are not well understood. Here, we investigated the function of miR-19a/b-3p/SIRT1/FoxO3/SPHK1 axis in ischemia/reperfusion (I/R).

**Methods:**

MCAO (middle cerebral artery occlusion) reperfusion rat model was used as the in vivo model of I/R. Cultured neuronal cells subjected to OGD/R (oxygen glucose deprivation/reperfusion) were used as the in vitro model of I/R. MTT assay was used to assess cell viability and TUNEL staining was used to measure cell apoptosis. H&E staining was employed to examine cell morphology. qRT-PCR and western blot were performed to determine levels of miR-19a/b-3p, SIRT1, FoxO3, SPHK1, NF-κB p65, and cytokines like TNF-α, IL-6, and IL-1β. EMSA and ChIP were performed to validate the interaction of FoxO3 with *SPHK1* promoter. Dual luciferase assay and RIP were used to verify the binding of miR-19a/b-3p with SIRT1 mRNA.

**Results:**

miR-19a/b-3p, FoxO3, SPHK1, NF-κB p65, and cytokines were elevated while SIRT1 was reduced in brain tissues following MCAO/reperfusion or in cells upon OGD/R. Knockdown of SPHK1 or FoxO3 suppressed I/R-induced inflammation and cell death. Furthermore, knockdown of FoxO3 reversed the effects of SIRT1 knockdown. Inhibition of the miR-19a/b-3p suppressed inflammation and this suppression was blocked by SIRT1 knockdown. FoxO3 bound SPHK1 promoter and activated its transcription. miR-19a/b-3p directly targeted SIRT1 mRNA.

**Conclusion:**

miR-19a/b-3p promotes inflammatory responses during I/R via targeting SIRT1/FoxO3/SPHK1 axis.

## Background

Stroke is the primary cause of morbidity and mortality worldwide and occurs when blood vessels in the brain get clogged, leading to insufficient oxygen and nutrient supply to brain tissues and subsequent cell death and brain injury [1]. Recovering the blood flow through drugs or mechanical interventions is key to recovery [2]. However, many studies have shown that the reperfusion will induce severe secondary injuries in that a large amount of cellular free oxygen radicals and inflammatory cytokines will be produced [3, 4]. Emerging evidence suggests that suppression of the inflammatory responses is beneficial for ischemic stroke [5]. Nevertheless, the underlying mechanisms of the reperfusion-induced inflammation are not well understood.

MicroRNAs (miRNAs) are a widely studied class of endogenous RNAs that are not translated into proteins but have crucial roles in many cellular processes including normal physiological and abnormal pathological processes [6, 7]. Many miRNAs have been implicated in the inflammatory responses during ischemic stroke [8, 9]. For instance, miR-22 has been shown to suppress the inflammation via targeting MAPK signaling [10]. miR-19a-3p/miR-19b-3p (miR-19a/b-3p) are important miRNAs that function as oncogenes to promote tumorigenesis and metastasis [11, 12]. Recent studies have reported that miR-19a/b-3p levels were elevated following ischemia/reperfusion (I/R) [13], suggesting that they might be involved in I/R. Nevertheless, the exact functions of miR-19a/b-3p in ischemic stroke are not clear. Sirtuin1 (SIRT1) is an NDA-dependent protein/histone deacetylase that play key roles in oxidative stress and inflammation via regulating various substrates [14]. Previous studies have indicated that SIRT1 alleviated the brain injury induced by I/R [15]. Further, SIRT1 could directly deacetylate FoxO3, a transcription factor, and negatively regulate FoxO3-induced transcription [16]. Through our preliminary bioinformatic analysis, we identified potential binding sites between miR-19a/b-3p and *SIRT1* mRNA. We thus hypothesized that miR-19a/b-3p function through targeting SIRT1/FoxO3 axis in ischemic stroke.

The sphingosine kinase 1 (SPHK1) is an enzyme that functions to phosphorylate sphingosine into sphingosine1 phosphate [17]. It is greatly involved in regulating cell proliferation, migration, and apoptosis [17]. Emerging evidence suggests that the SPHK1 has critical roles in mediating inflammatory responses under various conditions including I/R [18]. For example, the SPHK1 has been shown to activate NF-κB signaling to induce secretion of cytokines during I/R [19]. Our preliminary analysis through JASPAR revealed that FoxO3 might directly bind *SPHK1* promoter to regulate its transcription. Based on aforementioned literature and our initial analysis, we made a hypothesis that miR-19a/b-3p might be involved in I/R via SIRT1/FoxO3 which could regulate SPHK1-mediated neuroinflammation.

In the current study, we sought to test our above hypothesis and investigate the molecular mechanisms underlying the inflammatory responses during I/R, with a focus on miR-19a/b-3p/SIRT1/FoxO3/SPHK1 axis.

## Methods

### Middle cerebral artery occlusion (MCAO) rat model

All animal experiments and protocol have been reviewed and approved by the Animal Care and Use Committee of Southern Medical University. MCAO surgery was carried out as previously described [20]. Briefly, adult male Sprague Dawley (SD) rats (2–3 months old, 250 ~ 300 g b.w., *n* = 16) were anesthetized by intraperitoneally injecting ketamine and xylazine (250 and 10 mg/kg, respectively). The right common carotid artery (CCA) and external carotid artery (ECA) were dissected free and exposed. A surgical silica gel monofilament was introduced into the ECA until it passed the carotid bifurcation when a resistance was felt. The wound was closed after the monofilament was inserted. After 2 h of occlusion, the monofilament was withdrawn for reperfusion. In sham-surgery group, mice underwent similar procedures and the arteries were exposed for same duration without insertion of monofilament.

### Oxygen and glucose deprivation/reperfusion (OGD/R) cell model

To mimic neuronal injury during cerebral ischemia, human neuroblastoma cells (SK-N-SH, SH-SY5Y), purchased from Cell Bank of Type Culture Collection, Chinese Academy of Science (Shanghai, China), were subjected to oxygen and glucose deprivation followed by reperfusion (OGD/R). The cells were cultured in culture medium composed of Dulbecco’s modified Eagle’s medium (DMEM) (Gibco, CA, USA), 10% (vol/vol) fetal bovine serum (Gibco, CA, USA) and 1% penicillin-streptomycin. Cells were grown at 37 °C in the cell incubator with a humidified atmosphere (95%) containing 5% CO_2_. For OGD/R model, briefly, full culture medium was replaced with glucose-free DMEM, and the cells were placed in an anaerobic chamber (95% N_2_ and 5% CO_2_) at 37 °C for various periods (2, 4, 8, 12 h). Subsequently, full culture medium was added back and the cells were maintained in the normal culture condition.

### TTC staining

The infarct size was determined by TTC staining. Briefly, rats were anesthetized and the brains were immediately removed followed by coronally sectioning at 2 mm. Two percent 2,3,5-triphenyltetrazolium chloride (TTC) was added to incubate with the sections for 10 min at 37 °C to stain the infarct areas. Images were analyzed with ImageJ (National Institute of Health, USA, https://imagej.nih.gov/ij/) to quantify the infarct size by calculating the integration of infarct areas from all slices of the brain.

### H&E staining and TUNEL staining

Brain tissues were fixed in 10% formalin overnight at 4 °C, washed by PBS, and then embedded in paraffin. The tissues were cut into 5-μm-thick slices and then incubated with hematoxulin and eosin (H&E) or terminal deoxynucleotidyl transferase dUTP nick end labeling (TUNEL) staining reagents (Roche Applied Science, USA) for H&E staining or TUNEL staining, respectively, based on the manufacturer’s guidance. The stained brain sections were washed with PBS and then mounted with the mounting medium containing 4′,6-diamidino-2-phenylindole (DAPI). The percentage of apoptotic cells were calculated by dividing TUNEL-positive cells by DAPI-positive cells.

### Cell transfection

Lipofectamine 3000 (Invitrogen, USA) was used as the reagent for cell transfection as the manufacturer’s instruction describes. Briefly, cultured cells were cultured to ~ 80% confluence. The short hairpin RNAs (shRNAs) targeting SPHK1, SIRT1 and FoxO3, miR-19a/b-3p mimics, miR-19a/b-3p inhibitor, and corresponding negative controls were obtained from GenePharma (Shanghai, China). Corresponding constructs above together with Lipofectamine 3000 (m/v 1:1) were directly added into the culture media for 48 h followed by harvest.

### MTT cell proliferation assay

In total, 5000 transfected SK-N-SH or SH-SY5Y cells (as indicated in figure legends Figs. 2, 3, and 5) were plated into the single well of 96-well plates 24 h prior to 8 h of OGD/R and were followed by MTT (3-(4,5-dimethyltjiazol-2-yl)-2,5-diphenltetrazolium bromide) incubation (Abcam, USA). Fifty micrograms MTT was put into each well to incubate with cells for 3 h at 37 °C. Afterwards, 100 μl detergent reagent was used to stop the reaction. The absorbance in each condition was analyzed by a 490-nm wavelength.

### RNA immunoprecipitation (RIP) assay

MS2-binding sequences (MS2bs) were infused with binding sequences of FoxO3 in *WT-SIRT1* promoter or the mutant sequences. Cells were transfected with *MS2bs-SIRT1-WT, MS2bs-SIRT1-MUT*, or control vector MS2bs-Rluc together with MS2bp-GFP using lipofectamine 3000. After 2 days, transfected cells were lysed in lysis buffer (50 mM Tris-HCl, 180 mM NaCl, 2 mM EDTA, 1.5% NP-40, 1% sodium deoxycholate) containing RNase inhibitors and protease inhibitors (Sigma-Aldrich, MO, USA). Extracted proteins were incubated with relevant antibodies (anti-GFP1 or IgG as control) (Millipore, USA) overnight at 4 °C and then pulled down with protein G Sepharose beads (Millipore, USA). The beads were washed with lysis buffer first and then eluted with proteinase K (Sigma-Aldrich, MO, USA). The elution was proceeded for RNA purification with Trizol reagent (Invitrogen, MO, USA). Quantitative RT-PCR was performed to examine the RNA yield of target mRNAs. The primers are listed in the qRT-PCR section.

### Chromatin immunoprecipitation (ChIP) assay

ChIP was carried out with the commercial ChIP kit (Abcam, USA) as the manufacturer’s protocol described. Briefly, formaldehyde was used to cross-link proteins/DNA and cells were washed with PBS followed by harvest via micrococcal nuclease. Cell debris was removed through centrifugation, and the supernatant was collected. To pull down chromatin fragments, 10 μg of anti-FoxO3 or rabbit IgG antibody was added to incubate with the lysate for 1 h at 4 °C. Protein G beads were added to all samples for overnight incubation at 4 °C. The next day, the beads were washed by wash buffer and eluted by elution buffer. The elution was proceeded for DNA purification, and PCR was performed to detect *SPHK1* promoter region. The primers used for analysis were as follows: forward: 5′- CCT GGC GGC TTC TTT TTG TCC-3′; reverse: 5′- GGG GCT CTC ATC GGG ATT GG-3′.

### Electrophoretic mobility shift assay (EMSA)

FITC-labeled oligonucleotide probe corresponding to the FoxO3-binding site sequence in Sphk1 promoter and the mutant probe were synthesized and purchased from Genema (Shanghai, China). EMSA was performed by using the commercial EMSA kit (Thermo Fisher, USA). Native nuclear extracts were isolated from cells, and 5 μg protein extracts were incubated with relevant probes together with or without FoxO3 antibody in the binding buffer for 30 min at room temperature. EMSA loading dye was added to the reaction followed by electrophoresis in Tris-Glycine buffer. The signals of the FITC-labeled probes were detected using the ChemiDoc XRS+ system (Bio-Rad, USA).

### Dual-luciferase reporter assay

The Phusion Mutagenesis kit (Thermo Fisher, MA, USA) was used to mutate the binding sites as the manufacturer’s protocol described. cDNAs that included the wild-type sequences (*WT-SIRT1*) or mutated binding sequences (*MUT-SIRT1*) with miR-19a/b-3p in *SIRT1* 3′ UTR were cloned into the pGL4 luciferase reporter vector (Promega, WI, USA). Neuroblastoma cells were co-transfected with the recombinant plasmid together with miR-19a/b-3p mimics or mimics negative control (NC) (synthesized from Genepharma, Shanghai, China). Then, 24 h after, the co-transfected cells were harvested in the Reporter Lysis Buffer. The luciferase activity of each sample was measured using the Dual-Luciferase Reporter Assay System (Promega, WI, USA).

### RNA extraction and qRT-PCR

Trizol reagent (Invitrogen, Missouri, USA) was used to isolate total RNAs from cortex infarct tissues or cultured cells as the manufacturer’s instructions described. DNaseI was included into the lysis buffer to avoid the contamination of DNA. Then, 1–2 μg total RNA of each sample was used for reverse transcription and then amplified by PCR with standard kits (TaqPath RT-PCR Master mixes, Invitrogen, Missouri, USA). Relative expression levels of miRNA, *TNF-α*, *IL-1β*, and *IL-6* mRNAs were normalized U6 or GAPDH mRNA as internal controls, respectively. The primers used for the study were as follows:

miR-19a-3p forward primer: 5′-CGCTGTGCAAATCTATGCAAA-3′;

miR-19a-3p reverse primer: 5′-CAGTGTGCAAATCTATGCAA-3′;

miR-19b-3p forward primer: 5′-TGTGCAAATCCATGCAAAACTGA-3′;

miR-19b-3p reverse primer: 5′-CAGTGCGTGTCGTGGAGT-3′;

*TNF-α* forward primer: 5′-AGGCGCTCCCCAAGAAGACA-3′;

*TNF-α* reverse primer: 5′-TCCTTGGCAAAACTGCACCT-3′;

*IL-1β* forward primer: 5′-GCAGTCTACACAGCTTCGGG-3′;

*IL-1β* reverse primer: 5′-CCGCCTCAGCCTCCCAAAG-3′;

*IL-6* forward primer: 5′-GCCTTCGGTCCAGTTGCCTT-3′;

*IL-6* reverse primer: 5′-GCAGAATGAGATGAGTTGTC-3′;

*U6* forward primer: 5′-CTCGCTTCGGCAGCACA-3′;

*U6* reverse primer: 5′-AACGCTTCACGAATTTGCGT-3′;

*GAPDH* forward primer: 5′-GAGTCAACGGATTTGGTCGTT-3′;

*GAPDH* reverse primer: 5′-TTGATTTTGGAGGGATCTCG-3′.

### Western blot analysis

RIPA lysis buffer (Thermo Fisher, MI, USA) was utilized to extract proteins from rat brain cortex or cultured cells as previously described [20]. Protein concentration of each sample was measured by using Pierce™ BCA Protein Assay Kit (Thermo Fisher, MI, USA). Equal amounts of protein were loaded into SDS-polyacrylamide gels and separated via electrophoresis. Subsequently, the proteins in the gels were transferred to PVDF membranes (Sigma-Aldrich, USA). The membranes were first blocked with 3% BSA for half an hour at room temperature and then incubated with primary antibodies overnight at 4 °C. On the next day, the membranes were washed with TBST 3 times before incubation with specific secondary antibodies (Anti-Rabbit) for 1 h at room temperature. Signals were detected by using the standard ECL kit (Pierce ECL Kit, Thermo Fisher, USA). Primary antibodies used in the study were as follows: Rabbit polyclonal anti-SPHK1 antibody (1:1000, Abcam, USA); Rabbit polyclonal anti-NF-κB p65 antibody (1:1000, Abcam, USA); Rabbit monoclonal anti-FoxO3 antibody (1:1000, Cell Signaling, USA); Rabbit polyclonal anti-SIRT1 (1:1000, Abcam, USA); Rabbit polyclonal anti-β-actin (1:5000, Abcam, USA).

### Statistical analysis

All experiments were carried out with at least three biological replicates. All statistical analyses were analyzed in GraphPad Prism 7 (GraphPad, CA, USA). Unpaired Student’s t test (two groups) and one-way ANOVA (more than two groups) were used to determine the statistical significance (*P* < 0.05). The data were presented as mean ± SD (standard deviation).

## Results

### MCAO upregulated miR-19a/b-3p, FoxO3, and SPHK1, while it downregulated SIRT1

**Fig. 1:**
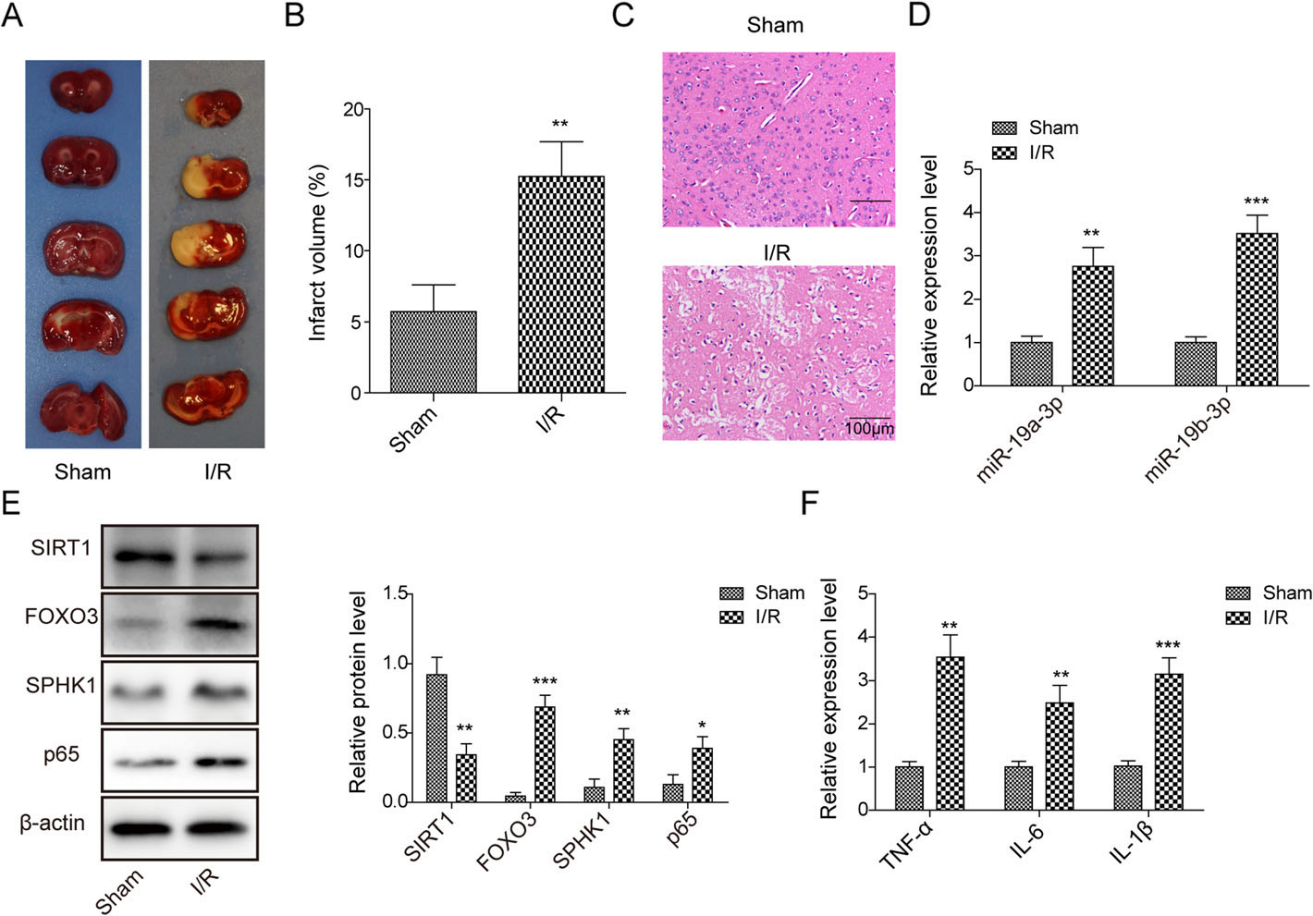
MCAO upregulated miR-19a/b-3p, FoxO3, and SPHK1, while downregulated SIRT1. **a** Representative images of TCC staining of brain sections from sham group or I/R group. **b** Quantifications of infarct volume in sham group and I/R group. **c** Representative images of H&E staining of brain sections from sham group or I/R group. **d** Relative miR-19a/b-3p levels in infarct tissues from sham group and I/R group. **e** Relative protein levels of SIRT1, FoxO3, SPHK1, and NF-κB p65 in infarct tissues from the sham group and I/R group. **f** Relative levels of inflammatory cytokines including *TNF-α*, *IL-6*, and *IL-1β* from sham group and I/R group. All the results were shown as mean ± SD (n = 3), which were three different experiments performed in triplicate. *P < 0.05, **P < 0.01, ***P < 0.01

To study the functions of miR-19a/b-3p, SPHK1, FoxO3, and SIRT1 in cerebral ischemia, we firstly measured their expression levels during ischemia/ reperfusion (I/R). MCAO surgery was performed in adult rats followed by reperfusion. As shown in Fig. 1a,b, I/R caused remarkable brain injury with obvious infarct area in the right hemisphere. H&E staining indicated robust neuronal degeneration following I/R compared to sham group (Fig. 1c). These results demonstrate the success of MCAO model. In infarct tissues, we observed an elevation of miR-19a/b-3p level compared to sham group (Fig. 1d). FoxO3, SPHK1, and NF-κB p65 protein levels were greatly upregulated, while SIRT1 downregulated (Fig. 1e). Besides, we found that MCAO also increased the levels of inflammatory cytokines including *TNF-α, IL-6*, and *IL-1β* (Fig. 1f). Taken together, these data show that I/R induced inflammation and neuronal injury, accompanied by an increase in expressions of miR-19a/b-3p, FoxO3, and SPHK1 and a decrease in SIRT1.

### Knockdown of SPHK1 suppressed OGD/R-induced cell death

**Fig. 2:**
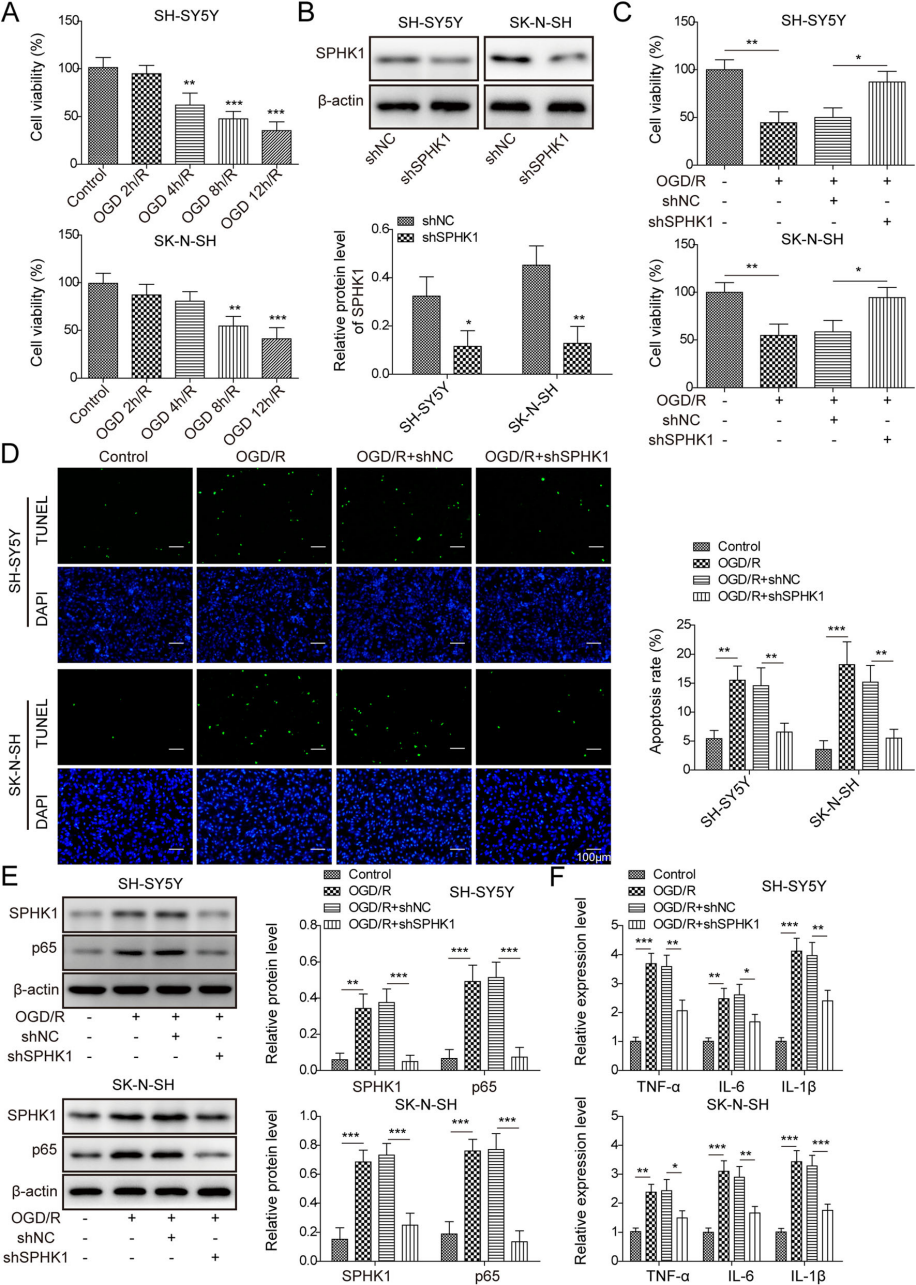
Knockdown of SPHK1 suppressed OGD/R-induced cell death. **a** MTT assay to analyze cell viability following various periods of OGD/R. **b** Relative SPHK1 protein level in cells transfected with sh-NC or sh-SPHK1. **c** MTT assay to analyze cell viability following OGD 8h/R in transfected cells. **d** TUNEL staining to measure the number of apoptotic cells in transfected cells upon OGD 8h/R. **e** Relative protein levels of SPHK1 and NF-κB p65 in transfected cells following OGD 8h/R. **f** Relative levels of *TNF-α*, *IL-6*, and *IL-1β* in transfected cells following OGD 8h/R. All the results were shown as mean ± SD (*n* = 3), which were three different experiments performed in triplicate. *P < 0.05, **P < 0.01, ***P < 0.01

To further investigate the function of SPHK1, we used the cell model of ischemia by subjecting cultured neuronal cells to oxygen and glucose deprivation/reperfusion (OGD/R). With MTT assay, as expected, OGD/R treatment greatly decreased the viability of cells in a time-dependent manner with bigger effect following longer period of treatment (Fig. 2a). We chose 8 h of OGD as the condition for subsequent studies. Transfection of cells with sh-SPHK1 robustly diminished the protein level (Fig. 2b). Notably, knockdown of SPHK1 in cells recovered the viability of cells upon OGD/R (Fig. 2c). Consistently, with TUNEL staining, we found OGD/R treatment drastically increased the number of apoptotic cells while knockdown of SPHK1 suppressed that increase (Fig. 2d). At the molecular level, we showed that OGD/R upregulated SPHK1 and NF-κB p65 protein levels, as well as inflammatory cytokines like TNF-α, IL-6, and IL-1β (Fig. 2e,f). However, transfection of cells with sh-SPHK1 partially restrained those increases (Fig. 2e,f). Altogether, these results suggest that knockdown of SPHK1 decreases OGD/R-induced cell injury and death.

### Knockdown of FoxO3 ameliorated OGD/R-induced cell death

**Fig. 3:**
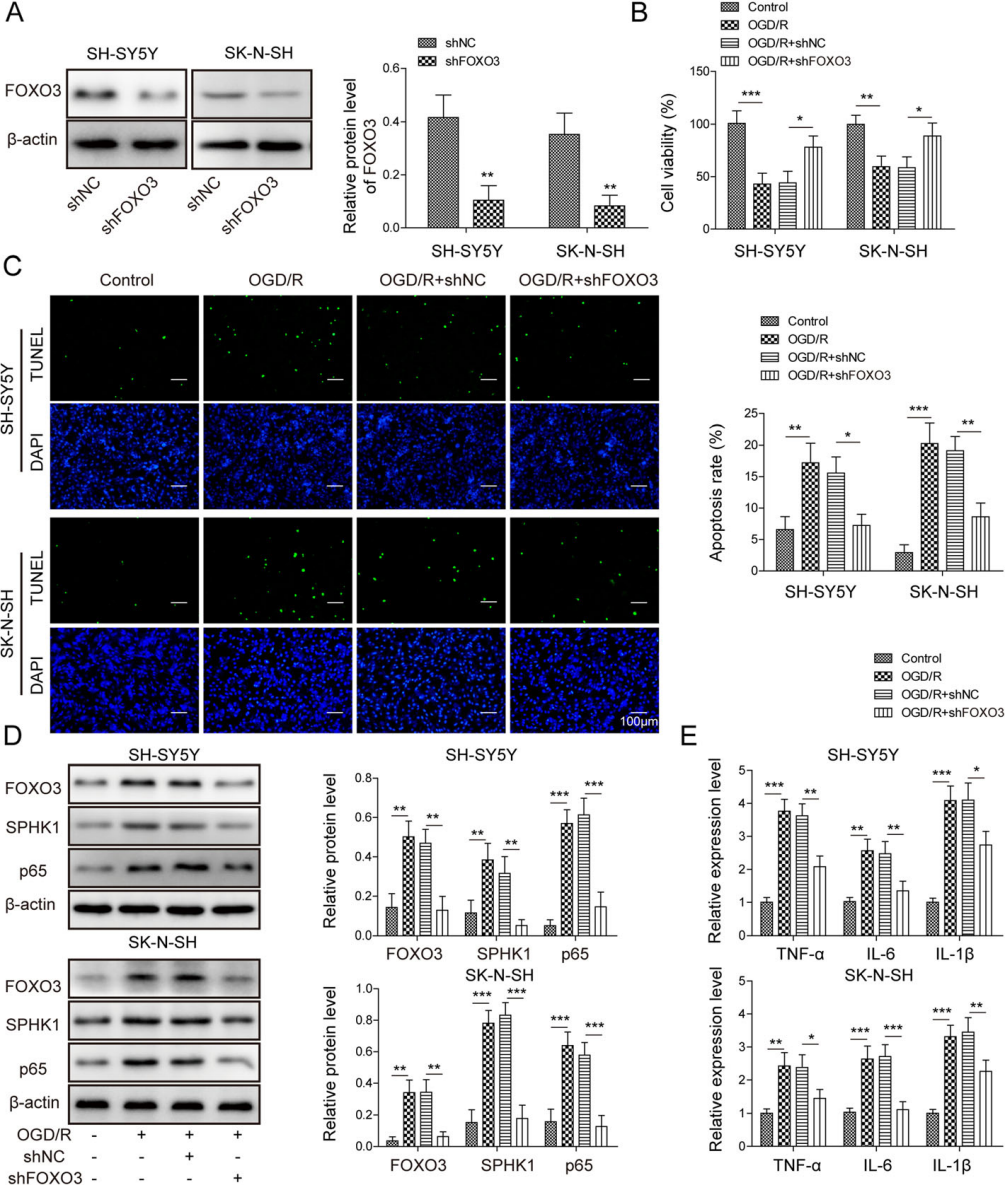
Knockdown of FoxO3 ameliorated OGD/R-induced cell death. **a** Relative FoxO3 protein level in cells transfected with sh-NC or sh-FoxO3. **b** MTT assay to analyze cell viability in transfected cells following OGD/R. **c** TUNEL staining to measure the number of apoptotic cells in transfected cells upon OGD/R. **d** Relative protein levels of SPHK1 and NF-κB p65 in transfected cells following OGD/R. **e** Relative levels of *TNF-α*, *IL-6*, and *IL-1β* in transfected cells following OGD/R. All the results were shown as mean ± SD (*n* = 3), which were three different experiments performed in triplicate. *P < 0.05, **P < 0.01, ***P < 0.01

We next examined the role of FoxO3 in ischemia and reperfusion. Knockdown of FoxO3 greatly decreased the level of FoxO3 in cells (Fig. 3a). With MTT assay, we found that knockdown of FoxO3 recovered the reduced viability of neuronal cells caused by OGD/R (Fig. 3b). Similarly, sh-FoxO3 suppressed the increased number of apoptotic cells upon OGD/R (Fig. 3c). Western blot results showed that OGD/R increased FoxO3 protein level while knockdown of FoxO3 repressed the elevated levels of FoxO3, SPHK1, and NF-κB p65 induced by OGD/R (Fig. 3d). In addition, the upregulation of cytokines such as TNF-α, IL-6, and during OGD/R were suppressed by sh-FoxO3 (Fig. 3e). We, therefore, conclude that knockdown of FoxO3 reduces OGD/R-induced cell death.

### FoxO3 transcriptionally activated SPHK1 expression

**Fig. 4:**
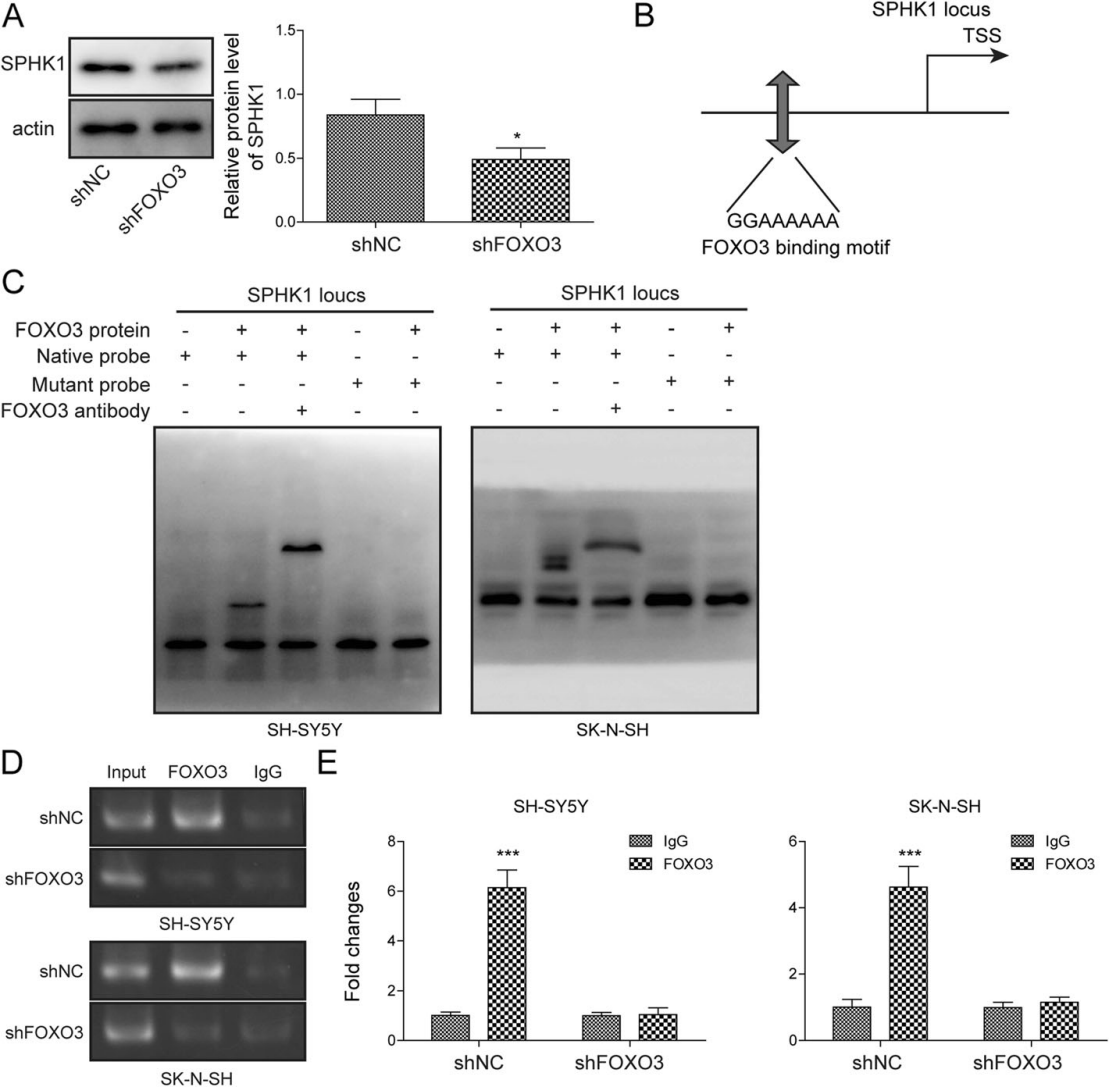
FoxO3 transcriptionally activated SPHK1 expression. **a** Relative SPHK1 protein levels in cells transfected with sh-NC or sh-FoxO3. **b** Predicted binding sites of FoxO3 in *SPHK1* promoter. **c** EMSA to determine the interaction between FoxO3 and *SPHK1* promoter. **d** ChIP to analyze FoxO3/*SPHK1* promoter interaction. **e**  Quantification of relative abundance of FoxO3 bound to *SPHK1* promoter. All the results were shown as mean ± SD (*n* = 3), which were three different experiments performed in triplicate. *P < 0.05, ***P < 0.01

FoxO3 is a transcription factor that regulates expression of multiple genes. We wondered whether FoxO3 modulated expression of SPHK1 since knockdown of both had similar effects on OGD/R-induced cell death. First, we found knockdown of FoxO3 in cells significantly decreased SPHK1 protein level (Fig. 4a). Through JASPAR analysis, we found a potential binding site of FoxO3 in the promoter region of *SPHK1* (Fig. 4b). To test whether FoxO3 directly bound *SPHK1* promoter, we employed the EMSA assay. As shown in Fig. 4c, we observed a DNA/protein complex when FoxO3 protein was incubated with the probe corresponding to the predicted FoxO3 binding region of *SPHK1* promoter, but not with the mutant probe wherein the binding sites were mutated. Furthermore, FoxO3 antibody further up-shifted the DNA/protein complex (Fig. 4c). Moreover, with ChIP, we found that immunoprecipitation with specific FoxO3 antibody significantly pulled down more *SPHK1* promoter compared to control IgG antibody (Fig. 4d). Knockdown of FoxO3 disrupted that interaction (Fig. 4d). These results provide evidence that FoxO3 directly binds the promoter of *SPHK1*.

### Knockdown of SIRT1 enhanced OGD/R-induced cell death via FoxO3

**Fig. 5:**
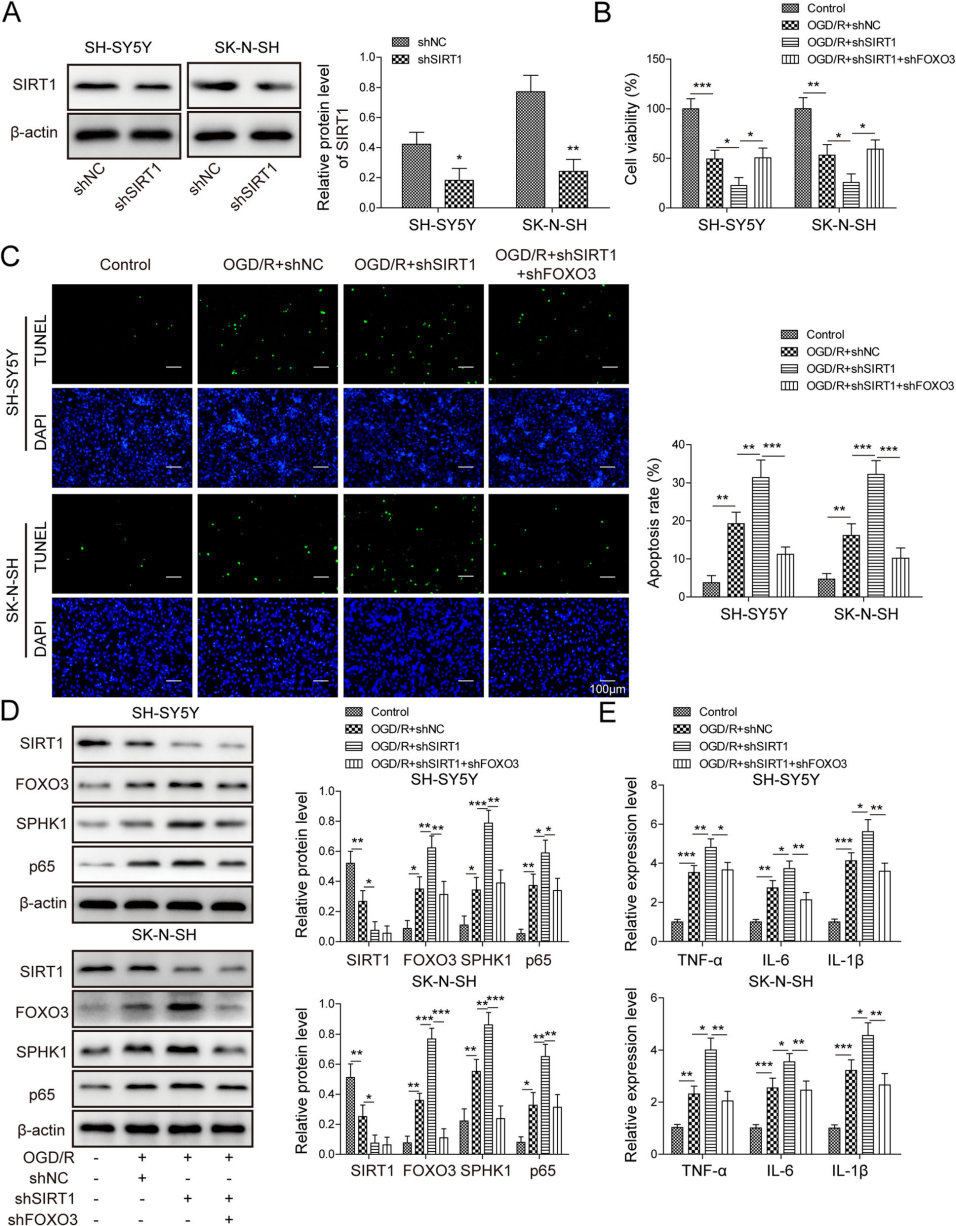
Knockdown of SIRT1 enhanced OGD/R-induced cell death via FoxO3. **a** Relative SIRT1 protein level in cells transfected with sh-NC or sh-SIRT1. **b** MTT assay to analyze cell viability in transfected cells following OGD/R. **c** TUNEL staining to measure the number of apoptotic cells in transfected cells upon OGD/R. **d** Relative protein levels of SIRT1, FoxO3, SPHK1, and NF-κB p65 in transfected cells following OGD/R. **e** Relative levels of *TNF-α*, *IL-6*, and *IL-1β* in transfected cells following OGD/R. All the results were shown as mean ± SD (*n* = 3), which were three different experiments performed in triplicate. *P < 0.05, **P < 0.01, ***P < 0.01

To study the role of SIRT1 in ischemia, we manipulated its level via shRNA and tested ensuing effects on cell death following OGD/R. Transfection of cells with sh-SIRT1 remarkably diminished the protein level of SIRT1 (Fig. 5a). With MTT assay, we showed that OGD/R deceased cell viability while knockdown of SIRT1 further reduced the viability (Fig. 5b). However, co-transfection of cells with sh-FoxO3 blocked the effects of sh-SIRT1 (Fig. 5b). We saw similar results in TUNEL staining. OGD/R greatly increased the number of apoptotic cells while knockdown of SIRT1 significantly further upregulated the number (Fig. 5c). Knockdown of FoxO3 suppressed the increase induced by SIRT1 knockdown (Fig. 5c). At the molecular level, we found that OGD/R decreased SIRT1 protein level (Fig. 5d). Knockdown of SIRT1 further decreased SIRT1 expression but increased the levels of FoxO3, SPHK1, and NF-κB p65 (Fig. 5d). Co-expression of sh-FoxO3 with sh-SIRT1 partially reversed those changes caused by sh-SIRT1 alone (Fig. 5d). Consistently, RT-qPCR results showed that knockdown of SIRT1 further upregulated levels of cytokines including TNF-α, IL-6, and IL-1β while sh-FoxO3 suppressed those increases caused by sh-SIRT1 (Fig. 5e). These data demonstrate that knockdown of SIRT1 promotes OGD/R-induced cell death via increasing FoxO3.

### The miR-19a/b-3p inhibitor suppressed OGD/R-induced cell injury via SIRT1

**Fig. 6:**
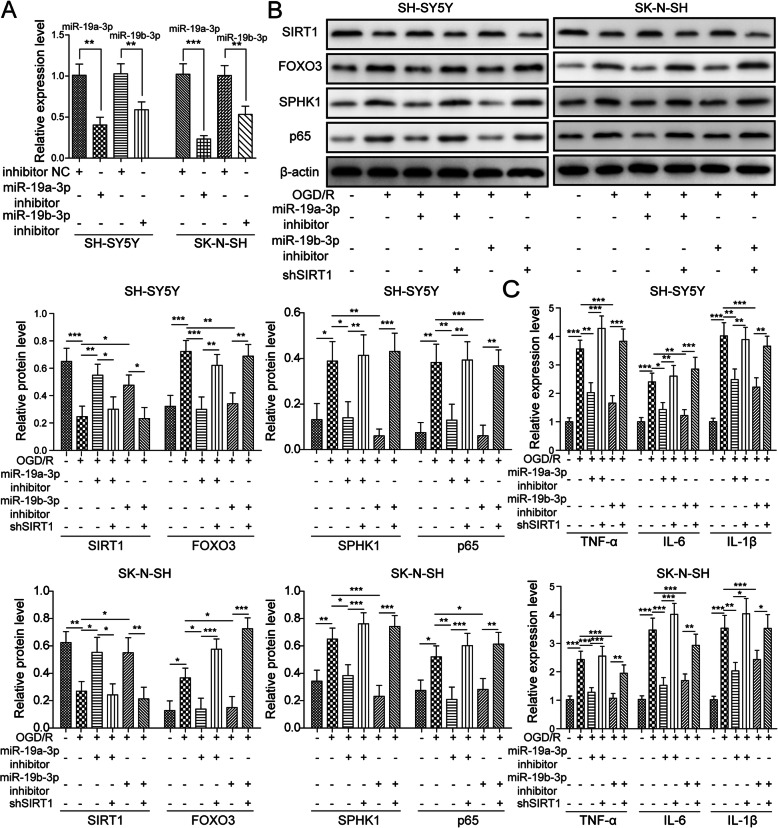
The miR-19a/b-3p inhibitor suppressed OGD/R-induced cell injury via SIRT1. **a** Relative miR-19a/b-3p levels in cells transfected with inhibitor NC or miR-19a/b-3p inhibitor. **b** Relative protein levels of SIRT1, FoxO3, SPHK1, and NF-κB p65 in transfected cells following OGD/R. **c** Relative levels of *TNF-α*, *IL-6*, and *IL-1β* in transfected cells following OGD/R. All the results were shown as mean ± SD (*n* = 3), which were three different experiments performed in triplicate. *P < 0.05, **P < 0.01, ***P < 0.01

We then investigated the functional role of miR-19a/b-3p in ischemia. As expected, transfection of cells with miR-19a/b-3p inhibitor robustly decreased miR-19a/b-3p level (Fig. 6a). Western blot results indicated that miR-19a/b-3p inhibitor reversed OGD/R-induced changes of protein expression by increasing SIRT1 level and decreasing the levels of FoxO3, SPHK1, and NF-κB p65 (Fig. 6b). Nevertheless, knockdown of SIRT1 with sh-SIRT1 reversed the effects of miR-19a/b-3p inhibitor, resulting in elevations of FoxO3, SPHK1, and NF-κB p65 (Fig. 6b). Similarly, the miR-19a/b-3p inhibitor suppressed the increases of cytokine levels such as TNF-α, IL-6, and IL-1β following OGD/R (Fig. 6c). Again, knockdown of SIRT1 blocked the effects of miR-19a/b-3p inhibitor (Fig. 6c). Together, these data indicate that miR-19a/b-3p inhibitor ameliorates OGD/R-induced cell injury via SIRT1.

### miR-19a/b-3p directly targeted SIRT1

**Fig. 7:**
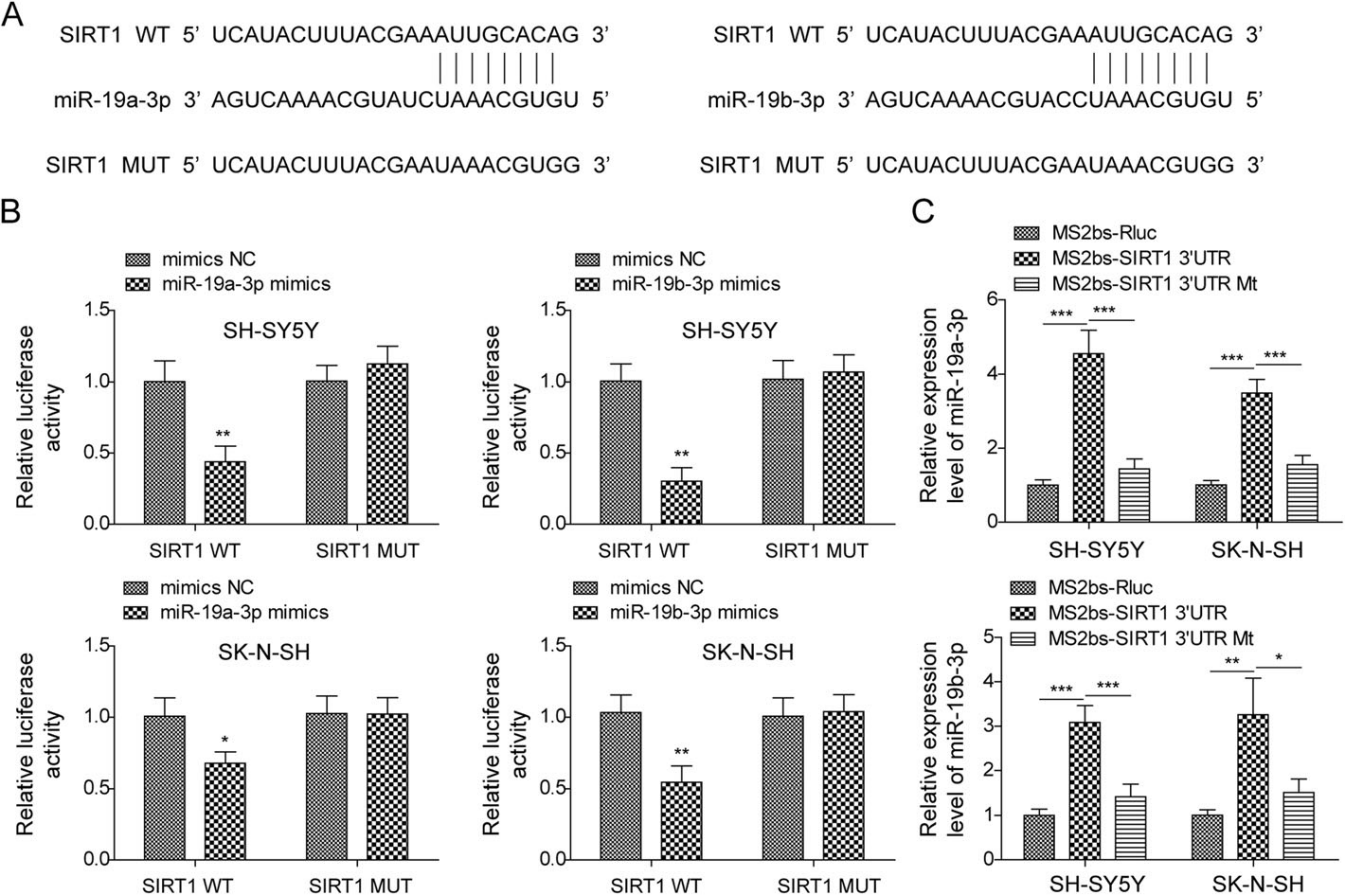
miR-19a/b-3p directly targeted SIRT1. **a** Predicted binding sites between miR-19a/b-3p and *SIRT1* mRNA and the sequences of *SIRT1* 3′UTR MUT. **b** Relative luciferase activities of *WT-SIRT1* 3′UTR and *MUT-SIRT1* 3′UTR in cells transfected with mimics NC or miR-19a/b-3p mimics. **c** RIP to analyze the binding between *WT-SIRT1* 3′UTR and miR-19a/b-3p. All the results were shown as mean ± SD (*n* = 3), which were three different experiments performed in triplicate. *P < 0.05, **P < 0.01, ***P < 0.01

Our aforementioned results show that miR-19a/b-3p negatively regulates SIRT1 expression, implying that SIRT1 might be a downstream target of miR-19a/b-3p. To test that, we first performed bioinformatic analysis by TargetScan and found some complementary binding sites between miR-19a/b-3p and *SIRT1* mRNA (Fig. 7a). To directly validate this interaction, we then used the dual luciferase assay and found miR-19a/b-3p mimics greatly decreased the luciferase activities of *WT-SIRT1* 3′UTR but not *MUT-SIRT1* 3′UTR wherein the predicted binding sites were mutated (Fig. 7b). Further, we used RIP assay to confirm the interaction in neuronal cells. Consistently, the result showed that immunoprecipitation of *WT-SIRT1* 3′UTR significantly enriched more miR-19a/b-3p compared to control of *MUT-SIRT1* 3′UTR (Fig. 7c). Altogether, these results demonstrate that miR-19a/b-3p directly binds *SIRT1* mRNA to negatively regulate its expression.

## Discussion

Stroke affects millions of people around the world and is the leading cause of disability and death [1, 21]. Despite numerous advances made, the mechanisms underlying reperfusion injury remain largely unknown and thus effective treatments are limited [2, 22]. In this study, we revealed that miR-19a/b-3p/SIRT1/FoxO3/SPHK1 axis play a crucial role in the inflammatory responses during I/R. miR-19a/b-3p, FoxO3, and SPHK1 were upregulated while SIRT1 was downregulated in I/R. Inhibition of miR-19a/b-3p or knockdown of FoxO3 and SPHK1 greatly suppressed the I/R-induced inflammation and cell death while knockdown of SIRT1 promoted them. Mechanistically, we showed that miR-19a/b-3p targeted SIRT1 while FoxO3 transcriptionally activated SPHK1.

SPHK1 is an important enzyme mediating the synthesis of sphingosine-1 phosphate [23]. SPHK1 has been shown to play critical roles in lipid metabolism, endoplasm reticulum stress, and mitochondrial function [24–26]. Further, SPHK1 is implicated in regulating inflammatory responses by activating NF-κB signaling or affecting releases of inflammation factors [19, 27]. Here, following I/R injury wherein inflammatory responses were initiated and cytokines were released, we observed an elevation of SPHK1 and induction of the NF-κB pathway. Moreover, knockdown of SPHK1 suppressed the levels of NF-κB p65 and cytokines including TNF-α, IL-6, and IL-1β, resulting in less cell death upon I/R. Our results demonstrate an essential role of SPHK1 in inducing inflammatory responses in I/R. Interestingly, we showed that I/R-induced elevation of SPHK1 was mediated by FoxO3-dependent activation of transcription. FoxO3 directly bound SPHK1 promoter and activated its transcription. Knockdown of FoxO3 had similar effects to SPHK1 knockdown. These results suggest that FoxO3/SPHK1 could serve as a promising target for suppression for ischemia treatment. FoxO3 is a very important transcription factor that regulates expression of many genes [28, 29]. Future studies are necessary to examine whether other targets of FoxO3 are involved in I/R. In addition, it remains to be explored whether the activation of FoxO3/SPHK1 signaling occurs during the ischemia phase or following reperfusion. Previous studies have reported an elevation of FoxO3 level during hypoxia [30, 31]. Therefore, it is possible that this pathway cascade is activated during ischemia and gets exaggerated following reperfusion.

As an NAD-dependent deacetylase, SIRT1 has various substrates and thus plays roles in many processes, such as cell proliferation/apoptosis, stress resistance, inflammation, and even autoimmunity [32–34]. Previous studies have shown that SIRT1 negatively regulates FoxO3 expression to modulate aging, skeletal muscle function, and cardiovascular homeostasis [35–38]. Consistently, SIRT1 level was inversely correlated with FoxO3 level in cells upon I/R. The upregulation of FoxO3 following I/R might be caused by reduced expression of SIRT1. Notably, we identified that *SIRT1* was a downstream target of miR-19a/b-3p. The decreased expression of SIRT1 following I/R was due to increased levels of miR-19a/b-3p. miR-19a/b-3p inhibitors suppressed the inflammation while knockdown of SIRT1 blocked that suppression. These data provide evidence that the interaction of miR-19a/b-3p and SIRT1 greatly contributes to I/R injury.

Both FoxO3 and SPHK1 have been implicated in myocardial I/R [38]. For instance, FoxO3 and SPHK1 were observed elevated during myocardial I/R [38, 39]. Furthermore, blockade of the activation ameliorated the cardiac inflammation and injury [18, 38]. In addition to that, reduced SIRT1 has been reported in cardiomyocytes during I/R injury and increasing SIRT1 level could confer protection against I/R injury in cardiomyocytes [40, 41]. Our study, together with previous studies, indicate that activation of FoxO3/SPHK1 is a conserved signaling pathway during I/R injury. Therefore, therapeutic strategies targeting FoxO3/SPHK1 could be widely used to combat I/R injury.

## Conclusions

In summary, in combination of both in vivo and in vitro models of ischemia/reperfusion, we demonstrate that miR-19a/b-3p/SIRT1 promotes neuroinflammation via regulating FoxO3/SPHK1 signaling, thus leading to cell death upon I/R. Suppression of the inflammation by targeting miR-19a/b-3p/SIRT1/FoxO3/SPHK1 could be a promising avenue to improve the outcome of ischemic stroke.

## Data Availability

All data generated or analyzed during this study are included in this article. The datasets used and/or analyzed during the current study are available from the corresponding author on reasonable request.

## Abbreviations

I/R: Ischemia/reperfusion
miRNAs: MicroRNAs
SIRT1: Sirtuin1
SPHK1: Sphingosine kinase 1
CCA: Carotid artery
ECA: External carotid artery
OGD/R: Oxygen and glucose deprivation followed by reperfusion
TTC: Triphenyltetrazolium chloride
H&E: Hematoxulin and eosin
TUNEL: Transferase dUTP nick end labeling
RIP: RNA immunoprecipitation
MS2bs: MS2-binding sequences
ChIP: Chromatin immunoprecipitation
EMSA: Electrophoretic mobility shift assay
WT-SIRT1: Wild-type sequences
MUT-SIRT1: Mutated binding sequences
SD: Standard deviation
NC: Negative control
